# A Comparative Study of Acidosis in Diabetic Advanced Chronic Kidney Disease Patients on and off Metformin

**DOI:** 10.7759/cureus.21291

**Published:** 2022-01-16

**Authors:** Ruqaya Qureshi, Kiran Nasir, Murtaza Dhrolia, Aasim Ahmad

**Affiliations:** 1 Nephrology, The Kidney Centre Post Graduate Training Institute, Karachi, PAK

**Keywords:** metformin associated lactic acidosis, diabetes mellitis, metabolic acidosis, metformin, chronic kidney disease

## Abstract

Aim

The aim of the study is to assess the risk of acidosis in diabetic advanced chronic kidney disease (CKD) patients on and off metformin.

Methods

This retrospective descriptive study was conducted in the nephrology department in The Kidney Centre Post Graduate Training Institute (TKC PGTI) Karachi from February to April 2020. We reviewed the records of all patients over 18 years old who visited the nephrology outpatient department in three months in 2020 (from February 2020 to April 2020), who had CKD (stage 2-5), are not on dialysis, and had type 2 diabetes. These were divided into two groups: those on metformin for more than one year and those not on metformin. We looked at hospitalizations due to acidosis in the previous one-year period.

Results

A total of 524 CKD patients had diabetes; out of those, 268 patients were on metformin, and 256 were not on metformin. The male vs. female distribution was 52.1% vs. 47.9%. A total of 114 (21.8%) patients required admission in the previous one-year period, and only 12 hospitalized patients had acidosis, seven (58.3%) were on metformin, and five (41.7%) were not on metformin, which was statistically insignificant.

Conclusion

Biguanides, especially metformin, is a known oral hypoglycemic drug used for decades to treat type 2 diabetes mellitus (DM). Metformin use is related to a rare but serious adverse event, metformin-associated lactic acidosis (MALA), especially in renal failure patients. In our study, metformin use in CKD diabetic patients did not result in more admissions due to acidosis than non-metformin users.

## Introduction

Globally one in 11 adults have diabetes mellitus (DM), more than 80% have type-2 diabetes, and among them, about 20% have an estimated glomerular filtration rate (eGFR) less than 60ml/min/1.73m^2^ [1]. Metformin hydrochloride is recommended as first-line therapy for glycemic control globally [2]. Metformin causes glucose-lowering effects by inhibiting gluconeogenesis and decreases the action of glucagon. The benefits of metformin besides glucose control include reducing blood pressure and plasma lipids, reducing body weight, and increase in insulin sensitivity [3].

Metformin is mainly associated with gastrointestinal side effects like nausea and vomiting and perceived risk of lactic acidosis (LA), a well known but rare side effect that mainly occurs in patients who have specific conditions like hypoxia, heart failure, renal failure, and sepsis, with an estimated incidence of 4.3 cases per 100,000 metformin users in a year [4]. Metformin-associated lactic acidosis (MALA) specifically refers to cases that cannot be explained by any significant risk factor other than metformin overdose [5]. Despite its global use, one of the areas of debate is whether metformin can be used in patients with renal impairment as the drug is excreted by the kidneys, with a potential of increased risk of developing acidosis and subsequently increasing the drug accumulation [6], but many think that this probability has significantly been overstated [7]. Regulatory and professional society proposed that this drug can only be used in mild to moderate chronic kidney disease (CKD) patients [8].

The recommendation for patients with eGFR between 30 and 60 ml/min has been a subject of discussion [9]. Various guidelines guardedly support metformin usage between eGFR of 30-60ml/min/1.73m^2^, endorsed that metformin is reviewed [10-11] at eGFR 45ml/min/1.73m^2^ and that dose adjusting should be regarded [12].

A detailed review of 347 trials by Salpeter et al. compared the LA incidence between those patients who were on metformin versus patients who were not on metformin in non-CKD patients, concluded that metformin is not associated with increased risk of LA [13]. In the diabetic CKD population in different stages, a study was done in 2017 using metformin with eGFR <60ml/min, compared with a similar group not on metformin concluded that there was no difference in plasma lactate levels in both groups [14].

In a retrospective study done among 77,601 patients to assess the frequency of MALA, participants were split into four groups based on their glomerular filtration rate (GFR): normal, mildly reduced, moderately reduced, or severely reduced. Results showed that there was no significance in the occurrence of MALA among these groups [15]. Another review article suggested a minor association between metformin and MALA, so metformin therapy can be used in those with deranged renal functions, but the dose should be adjusted for GFR [16].

## Materials and methods

This observational comparative descriptive study was carried out in the department of nephrology. An exemption was obtained from the ethical review committee of The Kidney Centre Post Graduate Training Institute (TKC-PGTI) Karachi. We reviewed the records of all adult patients who visited the nephrology outpatient department in the three-month period from February 2020 to April 2020, who had CKD (stage 2-5, not on dialysis), and who had type 2 diabetes. We reviewed and compared the hospital admissions of all the diabetic patients in the previous year due to acidosis on metformin (for more than a year) and those not on metformin.

A total of 1,500 patients visited the nephrology outpatient department in the three-month period. Diabetic patients were 662, out of which 138 were on hemodialysis (HD), and they were excluded. Of the 524 diabetic CKD patients, 268 were on metformin, and 256 were not on metformin. Duration of diabetes observed and dose and duration of metformin the patient was taking were noted. Demographic data, which included the age and sex of the patient, was also observed. Laboratory parameters were collected at baseline and during admission, i.e., serum bicarbonate, random blood sugar, serum creatinine, baseline glycosylated hemoglobin (HbA1c), arterial pH, and anion gap. The hospital admission date, discharge diagnosis, and patient outcome were also reviewed. The eGFR was calculated using the CKD Epidemiology Collaboration (CKD-EPI) formula, which included gender, age, serum concentration of creatinine, and race [17].

A CKD stage was allocated to each GFR estimation, following the Kidney Disease Improving Global Outcomes (KDIGO) classification (stage 1: eGFR ≥90 mL/min/1,73m^2^; stage 2: eGFR 60-89 mL/min/1,73m^2^; stage 3a: eGFR 45-59 mL/min/1,73m^2^; stage 3b: eGFR 30-44 mL/min/1,73m^2^; stage 4: eGFR 15-29 mL/min/1,73m^2^; stage 5: <15 mL/min/1,73m^2^) [18].

Five characteristics highly suggestive of MALA are severe acidemia (pH<7.1) with an anion gap greater than 20 meq/L (normal anion gap ≤12 mEq/L), very low serum bicarbonate (7 +/- 4 mEq/L), markedly elevated lactic acid (12.4 +/- 8 mmol/L), history of metformin ingestion, and history of renal insufficiency [19]. Besides lactate levels, we assessed all other parameters.

The data was entered and analyzed on SPSS version 21 (IBM Inc., Armonk, USA). Cleaning and coding of data were done before analysis. Mean ± STD and median with interquartile range were computed for continuous variables, while the frequency with percentages was calculated for categorical variables. Association between categorical variables was established by Chi-square test, independent t-test was applied for normally distributed continuous data, while Mann-Whitney U test was executed for skewed variables. The normality of data was checked by the Shapiro-Wilk test. A p-value of ≤0.05 was set as a significant level. 

## Results

Altogether 524 patients were recruited in our study - 268 patients were on metformin, and 256 were not on metformin, out of which 273 (52.1%) were male while 251 (47.9%) were female. The mean age was 57.7 ± 10.7, with a minimum of 26 and a maximum of 82 years. Out of 268 patients, 110 (41%) took metformin mostly from seven to 10 years, while 139 (51.9%) patients used 500mg twice daily. Among these diabetic patients, only 12 admissions occurred due to acidosis -seven (58.3%) were on metformin, and five (41.7%) were not on metformin. Demographic and clinical parameters of patients are presented in Table 1.

**Table 1:** Demographic and clinical parameters of diabetic patients (n=524) DM - diabetes mellitus; TKC - The Kidney Centre; CKD - chronic kidney disease; HCO_3_ - bicarbonate

Characteristics of patients		n (%)
Gender	Male	273 (52.1)
	Female	251 (47.9)
Duration of DM	1-3 years	3 (0.6)
	3.1-5 years	8 (1.6)
	5.1-7 years	33 (6.3)
	7.1-10 years	185 (35.3)
	>10 years	295 (56.3)
Duration of follow-up at TKC	<1 year	8 (1.5)
	1-3 years	60 (11.5)
	3.1-5years	104 (19.8)
	5.1-7 years	134 (25.6)
	7.1-10 years	166 (31.7)
	>10 years	52 (9.9)
Stage of CKD	2nd	5 (1)
	3rd	174 (33.2)
	4th	250 (47.7)
	5th	95 (18.1)
Patients on metformin		268 (51.1)
Duration of metformin use	1-3 years	3 (1.1)
	3.1-5 years	23 (8.6)
	5.1-7 years	54 (20.1)
	7.1-10 years	110 (41)
	>10 years	78 (29.1)
Dose of metformin	500mg od	16 (6)
	500 mg bd	139 (51.9)
	850 mg od	14 (5.2)
	850 mg bd	27 (10.1)
	1 gm od	1 (0.4)
	1 gm bd	9 (17.2)
	500 mg tds	46 (17.2)
	850+ 500 mg	16 (6)
Patients on HCO_3_ therapy		295 (56.3)
History of hospitalization in last one year		114 (21.8)
Cause of hospitalization	Acidosis	12 (10.5)
	Diarrhea	13 (11.4)
	UTI	48 (42.1)
	Obstruction	3 (2.6)
	Gastritis	5 (4.4)
	Malaria	3 (5.6)
	Hypoglycemia	5 (4.4)
	Pancreatitis	5 (4.4)
	Pneumonia	19 (16.7)

Laboratory parameters are described in Table 2.

**Table 2:** Age and laboratory parameters of patients HbA1c - glycosylated hemoglobin; HCO_3_ - bicarbonate; eGFR - estimated glomerular filtration rate; IQR - interquartile range

Variables	Mean ± STD	Median	IQR	Minimum	Maximum
Age	57.7 ± 10.7	59	14	26	82
HbA1c	8.1 ± 1.5	7.8	1.4	5	14
eGFR for the last three months	26.1 ± 11.5	24	17	8	66
Serum creatinine	2.7 ± 0.98	2.5	1.3	1.1	5.9
HCO_3_	21.4 ± 3.6	22	5	11	29
pH	7.4 ± 0.05	7.4	0.07	7.1	7.5

There was no association between gender, CKD stage, and history of hospitalization with metformin users and non-users. The patients who were using metformin had good control of diabetes, as shown by better HbA1c. Mean HbA1c of patients who were taking metformin was 7.8 ± 1.4 as compared to patients who were not using metformin (8.4 ± 1.5), and this difference is highly significant (p<0.001)

Similarly, the patients who were using metformin had high values of bicarbonate as compared to non-users (23 ±3 and 19.7± 3.4, respectively), and this difference is highly significant in the two groups (p<0.001). One hundred and six (39.6%) patients who were on metformin were on bicarbonate therapy, while 189 (73.8%) patients who were not on metformin were taking bicarbonate supplements (see Table 3).

**Table 3:** Association of characteristics of patients with metformin use HbA1c - glycosylated hemoglobin; HCO_3_ - bicarbonate; eGFR - estimated glomerular filtration rate; DM - diabetes mellitus; CKD - chronic kidney disease

Characteristics of all diabetic patients		Metformin use		p-value
		Yes	No	
Gender	Male	130 (47.6)	143 (52.4)	0.092
	Female	138 (55)	113 (45)	
Stage of CKD	2	5 (100)	0	0.154
	3	91 (52.3)	83 (47.7)	
	4	123 (49.2)	126 (50.6)	
	5	49 (51.6)	46 (48.4)	
History of hospitalization	Yes	51 (44.7)	63 (55.3)	0.122
	No	217 (52.9)	193 (47.1)	
Duration of DM	1-3 years	2 (66.7)	1 (33.3)	<0.001
	3.1-5 years	8 (100)	0	
	5.1-7 years	32 (97)	1 (3)	
	7.1-10 years	97 (52.4)	88 (47.6)	
	>10 years	129(43.7)	166 (56.3)	
On HCO_3_ therapy	Yes	106 (35.9)	189 (64.1)	<0.001
	No	162 (70.7)	67 (29.3)	
Age		56.9 ± 10.4	58.6 ± 10.8	0.061
HbA1c		7.8 ± 1.4	8.4 ± 1.5	<0.001
eGFR		26.5 ± 12.5	25.6 ± 10.5	0.855
Creatinine		2.7 ± 1.02	2.7 ± 0.94	0.621
HCO_3_		23 ± 3	19.7 ± 3.4	<0.001
pH		7.4 ± 0.05	7.4 ± 0.05	0.63

Table 4 shows the parameters of all hospitalized patients in the last year, either due to acidosis or other various reasons.

**Table 4:** Association of parameters of hospitalized patients with metformin use HCO_3_ - bicarbonate; RBS - random blood sugar; HD - hemodialysis

Parameters of hospitalized patients		Metformin use		p-value
		Yes	No	
Age		57.8 ± 11	58.7 ± 9.2	0.631
Total days of hospitalization		3.3 ± 1.2	2.9 ± 1	0.096
HCO_3_ at admission		19.2 ± 3.6	16 ± 4.3	<0.001
HCO_3_ at discharge		20.4 ± 3.2	17.8 ± 3.5	<0.001
Anion gap at admission		15.6 ± 1.9	13.6 ± 1.4	<0.001
Creatinine at admission		4 ± 1.7	3.8 ± 1.3	0.618
RBS at admission		169.5 ± 110.8	143.1 ± 70.8	0.97
Arterial HCO_3_		18.9 ± 3.3	15.3 ± 4.4	<0.001
Patients on HCO_3_ therapy	Yes	26 (35.1)	48 (64.9)	0.005
	No	25 (62.5)	15 (37.5)	
Cause of admission	Acidosis	7 (58.3)	5 (41.7)	0.463
	Diarrhea	6 (46.2)	7 (53.8)	
	UTI	18 (37.5)	30 (62.5)	
	Obstruction	3 (100)	0	
	Gastritis	2 (40)	3 (60)	
	Malaria	2 (66.7)	1 (33.3)	
	Hypoglycemia	4 (66.7)	2 (33.3)	
	Pancreatitis	2 (40)	3 (60)	
	Pneumonia	7 (36.8)	12 (63.2)	
Outcome of hospitalization	Discharge	43 (44.3)	54 (55.7)	0.919
	Needed HD	6 (50)	6 (50)	
	Death	2 (40)	3 (60)	

## Discussion

MALA occurs in patients taking metformin who have renal failure. There are different hypotheses regarding MALA: one theory is a sudden decrease in tubular secretion; this reduction is most commonly observed in acute renal failure, not in stable CKD patients. MALA is an unusual even; its predicted incidence is around 0.03 to 0.06/1,000 patients per year [20].

In our study, metformin was used in CKD stage 2 to 5 (not on dialysis) patients. Most of them were in stage 4 (n=123, 45.9%) followed by stage 3 (n=91, 34%) and stage 5 (n=49, 18.3%). Total 51 (44.7%) patients were admitted in the previous year, and out of them, only seven patients were admitted with acidosis. Apart from their CKD status, no additional apparent cause of acidosis was found other than the possibility of using metformin; out of seven patients, five patients were of stage 5 and two of stage 3 CKD. During admission, only one patient needed HD due to acidosis. Regarding non-metformin users, 126 (49.4%) patients were of stage 4, 83 (32.5%) were in stage 3, and 46 (18%) patients were in stage 5 (not on HD). In these patients, in terms of hospitalization, 63 (55.3%) patients were admitted in one year, and out of them, five patients were admitted due to acidosis in various stages of CKD. We did not find any significant difference in the risk of hospitalization due to acidosis in both groups.

Interestingly in metformin users, none of the patients of stage 5 CKD were admitted with acidosis in the designated period; this is consistent with two large retrospective trials on diabetic patients, most had a GFR of around 30ml/min but did not show an increase in hospitalization secondary to acidosis in those taking metformin [21]. Another study was done in Denmark on 10,652 diabetic patients (non-CKD), conducted for 14 years; 163 experienced an acute hospitalization due to LA corresponding to an increased rate of 391/100,000 person/years [22]. Although metformin is presumed to increase the risk of LA, we were unable to confirm this finding. The overall incidence of lactic acidosis in metformin users ranges from 3-47/100,000 person-years in the non-CKD population [8]. However, as far as the rate of hospitalization due to acidosis is concerned, we did not find any difference between CKD patients on metformin and those not on metformin (p-value 0.4).

DM itself causes increased concentration of lactate levels in the blood, so patients who are on metformin don't experience a predisposition to developing MALA [23].

Several large observational studies have traversed the connection between metformin and LA [24,25] but did not find any significant association.

In studies on patients continuing to receive metformin, even when they have GFR of less than 30-60ml/min, LA was a very rare complication, and risk for LA was similar to the risk seen with other agents in patients with similar degrees of renal impairment [26].

Ekstrom et al. studied 51,675 diabetic CKD patients and followed them for almost four years. They found that patients with eGFR between 45-60ml/min and metformin therapy had a lower risk of acidosis, severe infections, and all-cause mortality [27].

Overall the use of metformin for type 2 diabetic patients with decreased renal function remains controversial. One study showed metformin increases the risk of LA in patients with mild to moderate CKD, and this study highlighted that those patients who showed signs of dehydration or were on diuretics were more likely to develop MALA [28].

However, several studies showed no or minor association of MALA with metformin in renal failure patients, as revealed by Inzucchi et al. [8]. Their analysis showed that lactate levels were normal in patients with mild to moderate CKD who were on metformin therapy.

Data on safety in this population is scarce, so the recommendation for patients with GFR between 30-60ml/min has been a matter of debate [9]. In our study, most patients (139; 51.9%) took metformin 500mg in BID doses. Our study findings did not show any association of hospitalization with acidosis with different metformin dosages in CKD patients. Consistent with our study findings, Lalall et al. conducted a metformin dose-finding study in 78 patients with CKD stages 3 and 4, which concluded that suitable daily dosing does not increase the risk of LA in CKD patients [29]. In CKD patients, excretion of metformin is reduced so that a glucose-lowering effect can be achieved in a decreased dose of metformin. Dosage guidelines for CKD patients have been published, stating that 3gm for GFR 120ml/min, 2gm for GFR 60ml/min, 1gm for 30ml/min, 500mg for 15ml/min [30], but it would be advantageous to monitor therapy using regular metformin concentrations. Despite many conflicting theories, many physicians still prescribe metformin because metformin has multiple health benefits beyond its effect on glycemic control compared to other glycemic drugs.

This study is retrospective and done in a single renal center; we need to perform a prospective study to confirm the risk of acidosis in advanced chronic kidney patients with diabetes. 

## Conclusions

Metformin is used routinely in type 2 diabetic patients. Although MALA is a serious adverse event in CKD patients who are on metformin, in our study, we found no difference in one-year hospitalization with acidosis between CKD diabetic patients on or off metformin. Metformin is a biguanide, its most common side effects are related to the gastrointestinal tract like nausea, anorexia, bloating, and sometimes vomitings. MALA needs to keep in the differential diagnosis in chronic kidney disease patients who are on metformin to avoid the risk of severe consequences.
